# High Rates of *Mycobacterium tuberculosis* among Socially Marginalized Immigrants in Low-Incidence Area, 1991–2010, Italy

**DOI:** 10.3201/eid1909.120200

**Published:** 2013-09

**Authors:** Iacopo Baussano, Silvio Mercadante, Manish Pareek, Ajit Lalvani, Massimiliano Bugiani

**Affiliations:** International Agency for Research on Cancer, Lyon, France (I. Baussano);; UPO “A.Avogadro” and CPO–Piemonte, Novara, Italy (I. Baussano, S. Mercadante);; Imperial College, London, UK (M. Pareek, A. Lalvani);; Regional Reference Centre for Tuberculosis Prevention, Turin, Italy (M. Bugiani)

**Keywords:** tuberculosis, immigrants, latent tuberculosis, *Mycobacterium tuberculosis*, bacteria, Italy, tuberculosis and other mycobacteria, transmission, prevalence

## Abstract

Migration from low- and middle-income countries to high-income countries increasingly determines the severity of tuberculosis (TB) cases in the adopted country. Socially marginalized groups, about whom little is known, may account for a reservoir of TB among the immigrant populations. We investigated the rates of and risk factors for *Mycobacterium tuberculosis* transmission, infection, and disease in a cohort of 27,358 socially marginalized immigrants who were systematically screened (1991–2010) in an area of Italy with low TB incidence. Overall TB and latent TB infection prevalence and annual tuberculin skin testing conversion rates (i.e., incidence of new infection) were 2.7%, 34.6%, and 1.7%, respectively. Prevalence of both TB and latent TB infection and incidence of infection increased as a function of the estimated TB incidence in the immigrants’ countries of origin. Annual infection incidence decreased with time elapsed since immigration. These findings have implications for control policy and immigrant screening in countries with a low prevalence of TB.

Migration from low/middle income countries with high tuberculosis (TB) incidence increasingly accounts for most TB cases in high-income countries with low TB incidence; the greatest risk for active TB is within the first few years of arrival (*1*–*4*). Screening for active pulmonary TB when documented immigrants enter a new country has found ≈3.5 cases per 1,000 documented immigrants (*5*,*6*). The prevalence of smear-negative cases of TB reported for US-bound immigrants and refugees was 9.6 cases per 1,000 persons (*7*).

In countries with low incidence of TB, vulnerable populations, such as persons living in prisons (*8*) and shelters (*9*) and hard-to-reach populations (*10*–*13*), are at high risk for TB (*3*). Marginalized immigrants have the combined risk of coming from countries with high incidence of TB and being vulnerable because of their relegated social position in countries of destination (*14*). Recent evidence suggests that the distribution of latent TB infection (LTBI) and TB among immigrants is uneven. LTBI prevalence among recent immigrants to the United Kingdom increased as a function of TB incidence in the country of origin (*15*). In high-income countries, refugees, asylum seekers, and immigrants, who were screened for TB when entering the new country, had 11.9, 2.7, and 2.8 cases of TB/1,000 persons, respectively (*5*).

Among easy-to-reach immigrants, LTBI prevalence is around 40%, measured by tuberculin skin test (TST) (*16*,*17*), and 15%–19%, measured by interferon-γ release assays (IGRAs) (*11*,*16*). The largest study assessing TST results enrolled <1,000 undocumented immigrants (*17*); the studies that used IGRAs recruited no more than 125 undocumented immigrants (*11*,*16*). Estimates of prevalence of active TB among undocumented immigrants (range 0.0065%–1.6%) are based on <10 cases in each study (*11*,*16*). No data have been published on incidence of infection, the key indicator of *Mycobacterium tuberculosis* transmission, among socially marginalized immigrants. Knowledge of these parameters among socially marginalized groups could inform control strategies for TB in countries in which TB incidence is low.

We estimated the prevalence of and risk factors for active TB and LTBI, as well as the incidence of infection, among socially marginalized immigrants in an area of western Europe where incidence of TB is low. The study was conducted at the Regional Reference Centre for Tuberculosis Prevention in Turin, Italy. The estimated TB incidence during the study period, 1991–2010, was relatively stable (≈20 cases/100,000 persons), and in 2010, an increasing proportion of cases (≈70%) occurred among foreign-born persons (*18*–*20*).

## Methods

### Study Population

The immigrant population was recruited through a screening program designed to complement the National Health Service routine program to detect LTBI and TB in the general population, i.e., passive case finding and contact tracing of patients with active TB. The screening program was set up to systematically identify and test local persons and immigrants regardless of their documented status, as a mandatory prerequisite to access public and private health and social care facilities in Turin, such as shelters, canteens, charity-run outpatient clinics. According to national recommendations (*21*), these persons were tested for LTBI and, if clinically suspected, for active pulmonary TB. Because the social group is less likely to be detected through passive case finding and contact tracing, we defined this group of persons as a socially marginalized population. This population is usually made up of the homeless, drug abusers, and former prisoners and is recognized by the European Centre for Disease Prevention and Control as a group of persons who are particularly difficult to find and test and treat for LTBI and active TB (*22*).

Details of the screening procedures were reported elsewhere (*17*). In brief, LTBI was diagnosed by means of a TST performed by using intradermal injection of PPD-5IU (Biocine Sclavo, Siena, Italy). If active pulmonary TB was suspected, chest radiography was performed at the first consultation. Patients were then asked to return for a second consultation at which the TST result would be read and chest radiography performed for those with a positive TST result (i.e., induration diameter at least 10 mm). Patients with suspect TB on the basis of a chest radiograph were further tested by smear examination and culture. All patients with suspected TB who were capable of producing sputum had at least 3 sputum specimens submitted for microscopic examination for acid-fast bacilli detection and culture. Since 1988, the culture methods used were BACTEC radiometric blood culture system (Johnston Laboratories, Inc., Towson, MD, USA) and after 1998, MGIT 960 (Becton Dickinson Diagnostic Instrument Systems, Sparks, MD, USA) and confirmed by growth on conventional Lowenstein-Jensen media. Finally, patients with a TB diagnosis were treated with standard short-course chemotherapy, whereas patients with no abnormalities on chest radiographs and positive TST results were offered preventive treatment (*23*). Patients who moved into or transferred between social care facilities were rescreened. Regional TB surveillance programs provided quality assurance and regular training of screening personnel (*24*). No patients with known contact with a person with active TB were included in the analysis. We have analyzed the data collected during January 1991–December 2010. The set information included in the analyzed dataset is reported in the online Technical Appendix (Technical Appendix).

The geographic origin of each patient was categorized according to TB incidence data from the World Health Organization (WHO). In particular, each patient was assigned to one of the following categories: very low incidence (<25 annual cases/10^5^ population), low incidence (25–49 annual cases/10^5^ population), intermediate incidence (50–99 annual cases/10^5^ population), high incidence (100–299 annual cases/10^5^ population), and very high incidence (>300 cases/10^5^ population). The estimated annual incidence for each country at specific year of immigration was obtained from the WHO Global Atlas (*25*). If data relative to the specific year of immigration were not available, we considered the closest available year. For the annual incidence of active TB in the former Soviet Union, before 1995, we used the estimates reported by WHO (*26*).

### Case Definitions

Cases of active pulmonary TB were defined according to WHO and International Union Against Tuberculosis and Lung Disease recommendations (*23*). Cases were defined as microbiologically confirmed or “definite cases” (i.e., sputum smear examination positive for acid-fast bacilli or culture positive for *M. tuberculosis* complex) and “other than definite” cases (i.e., with negative smear sputum and missing or without culture examination but with radiographic and/or clinical picture consistent with TB). TB cases diagnosed at first visit were considered as prevalent cases. Patients with cutaneous induration of at least 10 mm diameter at 48–72 hours after TST inoculation, normal chest radiographs, and absence of symptoms were considered to have prevalent cases of LTBI, regardless of bacillus Calmette–Guérin (BCG) vaccination history. We considered alternative methods of interpreting TST results (*27*), but given the highly heterogeneous nature of our population in terms of geographic origin (>100 countries), the predominance of prior BCG vaccination, and the need for a well-defined TST cutoff for clinical decision-making, we adopted the above cutoff as recommended by national (*21*) and international guidelines (*28*). Finally, incident LTBI was defined as TST conversion in the absence of active TB; TST conversion was defined as an increase of at least 10 mm from the previous negative TST result in accordance with national (21) and international guidelines (*29*).

### Statistical Analysis

We describe the crude distribution of prevalent and incident LTBI cases and prevalent active pulmonary TB cases by using the following variables: sex, estimated annual TB incidence rate in the country of origin of the immigrants, age at first test, age at immigration, and year of immigration. The 95% CIs of LTBI and TB prevalence were calculated by assuming a binomial distribution of the prevalent cases and a Poisson distribution of the LTBI incident cases.

To assess the role of selected determinants on the risk for LTBI and TB occurrence, we performed both univariate and multiple regression analyses. In particular, using logistic models we tested the mutually adjusted effect of sex, estimated annual TB incidence rate in the country of origin of the immigrants, and age at test and time elapsed since immigration as determinants of LTBI and TB prevalence. Incidence of *M. tuberculosis* infection was estimated for immigrants who had had a second TST performed and read at least 1 year after the initial TST; each of these immigrants whose TST result converted from negative to positive was deemed to have acquired infection during the time between the TSTs. Finally, we tested the interaction between sex and the other model covariates.

## Results

**Figure 1:**
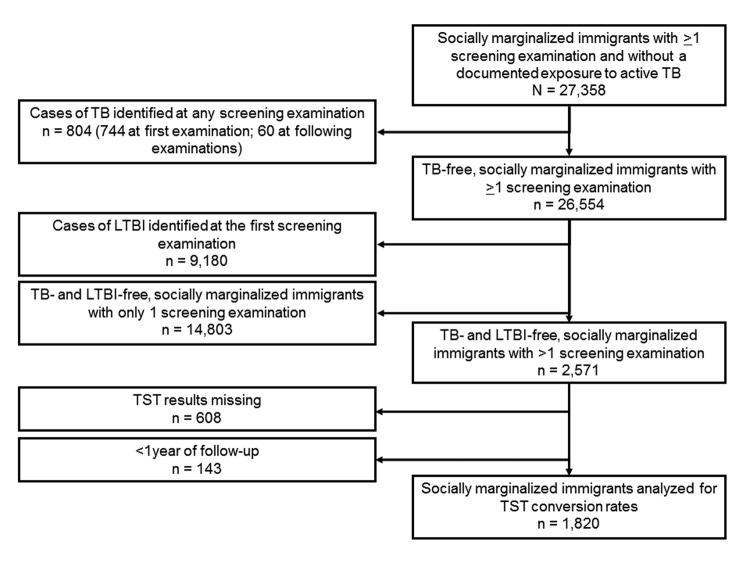
Immigrant selection process. TB, tuberculosis; LTBI, latent TB infection; TST, tuberculin skin testing.

From 1991 through 2010, a total of 27,358 socially marginalized immigrants who attended the screening program in Turin were considered at risk for active pulmonary TB. A total of 804 cases of TB (557 [69%] definite cases and 247 [31%] other than definite cases) were identified through the screening program, 744 (93%) of which were diagnosed at first visit. The prevalence of LTBI and incidence of TB among the remaining 26,554 immigrants who did not previously have TB were estimated with at least 1 and 2 valid screening examinations, respectively, (Figure 1).

Table 1 summarizes the main characteristics of the study population. Nearly 60% of the socially marginalized immigrants screened were men (n = 16,242), and 75% (n = 20,050 [73.3%]) were from countries with a high estimated annual incidence of TB (i.e., 100–299 cases/100,000 population); nearly 70% of the immigrants were 20–40 years of age at the time of immigration and screening. More than 80% entered Italy and were tested before 2005. The cumulative fraction of immigrants tested was 60%, 82%, and 95%, within 2, 5, and 10 years since immigration, respectively. Finally, the median interval between first and second TSTs for socially marginalized immigrants who had 2 TSTs and whose 1st TST result was negative was 5.6 years (interquartile range 2.9–8.6); for 143 immigrants, the interval was >1 year. Further details about the geographic origin of the immigrants can be found in the online appendix (Technical Appendix, Table 2).

**Table 1:** Descriptive characteristics of socially marginalized immigrants investigated, Italy*

Variable, group	No. (%)	
TB incidence rate in the country of origin of the immigrants, (x 10^5^ person-years)		
<25	679 (2.5)	
25–49	3,500 (12.8)	
50–99	1,668 (6.1)	
100–299	20,050 (73.3)	
>300	1,461 (5.3)	
Age at test		
<20	2,500 (9.1)	
20–29	10,705 (39.1)	
30–39	8,659 (31.7)	
>40	4,938 (18.0)	
Missing	556 (2.0)	
Age at immigration		
<20	3,416 (12.5)	
20–29	12,415 (45.4)	
30–39	6,318 (23.1)	
>40	4,778 (17.5)	
Missing	431 (1.6)	
Sex		
F	11,116 (40.6)	
M	16,242 (59.4)	
Year of immigration		
<1990	1,978 (7.2)	
1990–1994	5,121 (18.7)	
1995–1999	8,206 (30)	
2000–2004	8,911 (32.6)	
2005–2010	2,092 (7.6)	
Year of first testing		
1991–1994	2,040 (7.5)	
1995–1999	10,783 (39.4)	
2000–2004	11,400 (41.7)	
2005–2010	3,055 (11.2)	
First TST within		
2 y	60.4 (59.8–61)	
5 y	81.9 (81.4–82.3)	
10 y	95 (94.8–95.3)	

*TB, tuberculosis; TST, tuberculin skin test. Given missing data the totals do not always sum up to 100%.

**Table 2:** Distribution and prevalence/rate of TB and LTBI cases, and LTBI conversions*

	TB			LTBI				
Variable	No. cases/population at risk	Prevalence, % (95% CI)		No. cases/population at risk	Prevalence, % (95% CI)		No. cases/p-y at risk	Conv. rates, % (95% CI)
Total	744/27,358	2.7 (2.5–2.9)		9,180/26,554	34.6 (34.0–35.1)		90/5,241	1.7 (1.4–2.1)
Sex								
F	254/11,116	2.3 (2.0–2.6)		3,663/10,849	33.8 (32.9–34.7)		46/2,462	1.9 (1.4–2.5)
M	490/16,242	3.0 (2.8–3.3)		5,517/15,705	35.1 (34.4–35.9)		44/2,777	1.6 (1.2–2.1)
Age, y, at test								
<20	30/2,498	1.2 (0.8–1.7)		405/2,465	16.4 (15.0–17.9)		4/245	1.6 (0.1–4.3)
20–29	190/10,705	1.8 (1.5–2.0)		3,163/10,500	30.1 (29.2–31.0)		26/1,436	1.8 (1.2–2.7)
30–39	253/8,659	2.9 (2.6–3.3)		3,480/8,382	41.5 (40.5- 42.6)		41/2,301	1.8 (1.3–2.4)
>40	271/5,414	5.0 (4.4–5.6)		1,889/4,671	40.4 (39.0–41.9)		19/1,258	1.5 (1.0–2.4)
TB incidence (cases/10^5^ p-ys) in immigrant’s country of origin								
<25	4/679	0.6 (0.2–1. 6)		111/674	16.5 (13.9–19.5)		1/74	1.3 (0.2–9.6)
25–49	56/3,500	1.6 (1.2–2.1)		1,008/3,440	29.3 (27.8–30.8)		6/814	0.7 (0.3–1.6)
50–99	48/1,668	2.9 (2.2–3.8)		407/1,615	25.2 (23.1–27.4)		3/311	1.0 (0.3–3.0)
100–299	602/20,050	3.0 (2.8–3.2)		7,196/19,400	37.1 (36.4–37.8)		74/3,764	2.0 (1.6–2.5)
>300	34/1,461	2.3 (1.7–3.2)		458/1,425	32.1 (29.8–34.6)		6/278	2.1 (1.0–4.8)

*TB, tuberculosis; LTBI, latent TB infection; p-y, person years; conv., conversion.

**Figure 2:**
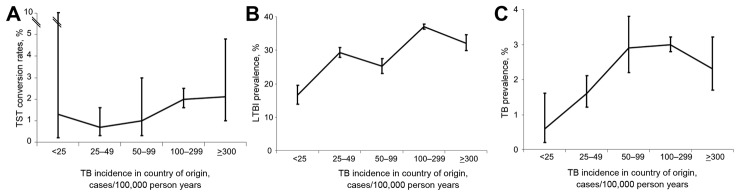
TB and LTBI among socially marginalized immigrants in an area of Italy where incidence of TB is low, by tuberculosis incidence rate in their country of origin, 1991–2010. A) TST conversion rates. B) LTBI prevalence. C) TB prevalence. Vertical bars indicate 95% CIs. TB, tuberculosis; LTBI, latent TB infection; TST, tuberculin skin testing.

The distribution of TB and LTBI cases and TST conversions by sex, age at testing, and incidence rate of TB in the country of origin of the immigrants is reported in Table 2 along with crude estimates (and 95% CIs) of TB prevalence, LTBI prevalence, and infection incidence rates. The overall prevalence of active pulmonary TB was 2.7% (2.5%–2.9%), LTBI prevalence at first TST was 34.6% (34.0%–35.1%), and annual infection incidence was 1.7% (1.4%–2.1%). The prevalence of TB and LTBI among men was higher than that among women, 3.0% (2.8%–3.3%) vs. 2.3 (2.0%–2.6%) and 35.1% (34.4% –35.9%) vs. 3.8% (32.9%–34.7%), respectively (Table 2). However, the difference in infection incidence between men and women was not statistically significant, 1.6% (1.2%- 2.1%) and 1.9% (1.4–2.5%), p = 0.5, respectively (Table 2). As expected, the prevalence of TB and LTBI increased as a function of age at the time of testing, whereas the infection incidence rates did not appear to be affected by age at the time of testing. Finally, the overall distribution of the TB and LTBI cases suggests that TB and LTBI prevalence and infection incidence rates increased as a function of TB incidence rate in the country of origin of the immigrants (Figure 2). We have provided additional tables showing microbiologically confirmed cases of TB (Technical Appendix, Tables 4 and 5).

**Figure 3:**
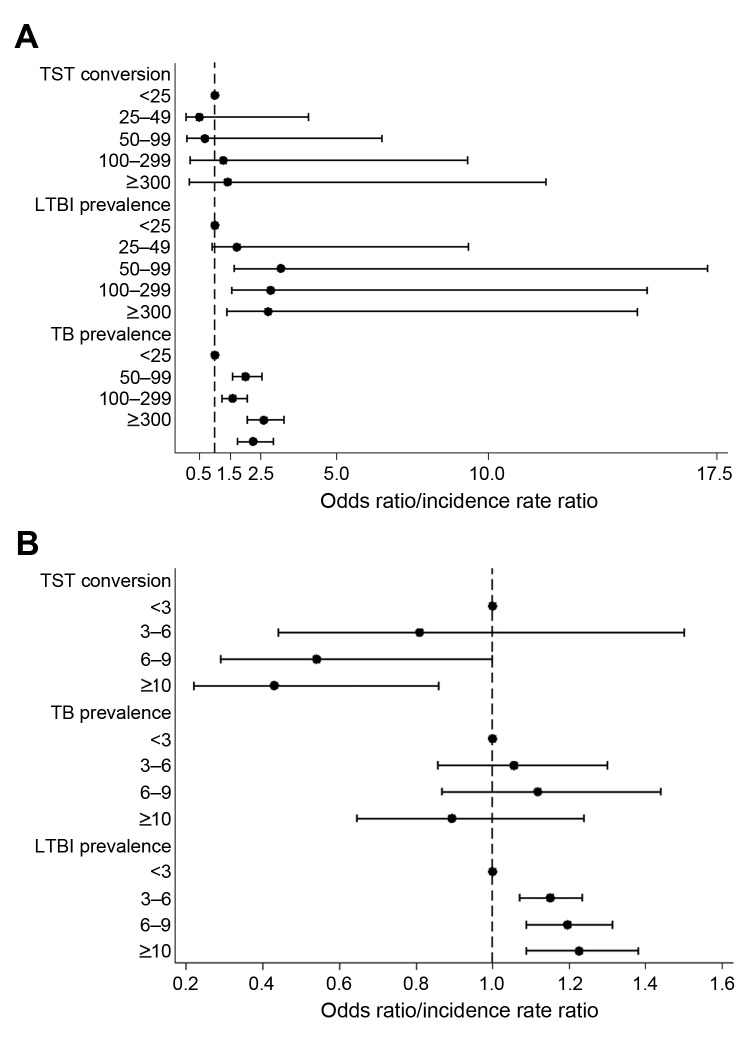
Adjusted odds ratio and incidence rate ratios for TB and LTBI occurrence among socially marginalized immigrants in a low-incidence area of Italy, by TB incidence rate in the country of origin (A) and time elapsed since immigration (B), 1991–2010. *Indicates reference category. Horizontal bars indicate 95% CIs. TB, tuberculosis; LTBI, latent TB infection; TST, tuberculin skin testing.

The crude and adjusted effects of sex, estimated TB incidence rate in the country of origin of the immigrants, age at testing, and time elapsed since immigration on TB and LTBI prevalence and TST conversion are reported in the Technical Appendix, Table 3, and Table 3 in this article. The odds ratio for TB and LTBI and the incidence rate ratio for TST conversion increased as a function of TB incidence rate in the country of origin of the immigrants (Figure 3, panel A). In particular, considering immigrants from countries with an estimated TB incidence of <25 cases/10^5^population as a reference category, the odds ratio for TB reached a plateau, ranging from 4.6 to 5.3, as the estimated TB incidence rate in the country of origin of the immigrants was >50 TB cases/10^5^ population (Table 3). The odds ratio for LTBI prevalence in immigrants from countries with an incidence in the country of origin >25 annual cases/10^5^ person-years was higher than that observed in the reference category, although no specific patterns could be identified (Table 3). The estimated TB incidence rate in the country of origin of the immigrants displayed a linear effect on the incidence rate ratio for LTBI conversion with an incidence rate ratio of 1.2 (1.1–1.4) for an increase of 50 cases/10^5^ person-years; such an effect was not discernible when the categorical definition of estimated TB incidence was used (Figure 3, upper panel). As observed for crude estimates reported in Table 2, men were at a higher risk than women for TB and LTBI (odds ratio 1.5 [1.3–1.8] and 1.1 [1.0–1.2], respectively); this difference disappeared when the incidence rate ratio for infection incidence (0.9 [0.6–1.3]) was considered. Similarly, age at testing displayed a linear and more than linear effect on the risk for TB and LTBI, respectively, whereas it did not show any effect on the LTBI conversion rates. The risk for TB and LTBI and TST conversion rate did not change and increased and decreased as a function of time elapsed since immigration (Figure 3, panel B). No interaction between sex and the other model covariates was observed.

**Table 3:** Multivariate model (logistic and Poisson) estimates of the odds and incidence rate ratios for TB, LTBI prevalence, and LTBI conversion*

Variable	Odds ratio (95% CI) for prevalence		Incidence rate ratio (95% CI) for TST conversion
	TB	LTBI	
TB incidence rate in the country of origin of the immigrants (per 10^5^ p-y)			
<25	1 (reference category)	1 (reference cat)	1 (reference category)†
25–49	2.9 (0.9–9.3)	2.0 (1.6–2.6)	0.5 (0.1–4.1)†
50–99	5.3 (1.6–17.2)	1.6 (1.2–2.1)	0.7 (0.1–6.5)†
100–299	4.9 (1.6–15.2)	2.6 (2.1–3.3)	1.3 (0.2–9.3)†
>300	4.6 (1.4–14.9)	2.2 (1.7–2.9)	1.4 (0.2–11.9)†
Sex			
F	1 (reference category)	1 (ref category)	1 (reference category)
M	1.5 (1.3–1.8)	1.1 (1.0–1.2)	0.9 (0.6–1.3)
Time elapsed since immigration, y			
<3	1 (reference category)	1 (ref cat)	1 (reference category)
3–6	1.1 (0.9–1.3)	1.1 (1.0–1.2)	0.8 (0.4–1.5)
7–9	1.1 (0.9–1.3)	1.2 (1.1–1.3)	0.5 (0.3–1.0)
>10	0.9 (0.6–1.2)	1.2 (1.1–1.4)	0.4 (0.2–0.9)
Age, y, at test			
<20	1 (reference category)	1 (reference category)	1 (reference category)
20–29	1.4 (1.0–2.2)	1.9 (1.7–2.2)	1.0 (0.4–3.0)
30–40	2.4 (1.6–3.5)	3.1 (2.8–3.5)	1.2 (0.4–3.5)
>40	4.3 (2.9–6.4)	3.1 (2.7–3.5)	1.0 (0.3–3.1)

*The effects of the variables reported in the table were mutually adjusted. TB, tuberculosis; LTBI, latent TB infection; p-y, person-years. †Incidence rate ratio for tuberculin skin test conversion (95% CI): 1.21 (1.07–1.36) for an increase of 50 cases/105 p-y.

## Discussion

Our study shows that socially marginalized immigrant populations as a whole may act as a reservoir for *M. tuberculosis*. In particular, the number of cases of TB and LTBI among socially marginalized immigrants, as measured through prevalence, was much higher than that reported among other immigrants or socially marginalized subgroups (*5*,*6*,*10*,*12*). Furthermore, the estimated TST conversion rates indicate that considerable *M. tuberculosis* transmission is occurring among socially marginalized immigrants in their adopted countries.

The estimated prevalence of active TB cases (2.7%), 69% of which were microbiologically confirmed (Technical Appendix, Table 4), is considerably higher than the estimated incidence rates among in the general population in the respective different countries of origin. This finding may reflect demographic and socioeconomic differences between socially marginalized immigrants and the general population in the countries of origin, such as age distribution and the poor social and living conditions of socially marginalized immigrants. Undocumented immigrants may also be less likely to seek medical attention. The estimated TB prevalence is higher than that found when screening for active pulmonary TB among refugees at entry into the new country (1.2%) (*5*). The prevalence of microbiologically confirmed cases (1.8%) is also higher for this population than for the homeless populations in London (*10*) and Rotterdam (*12*) (0.8% and 0.9%, respectively). The TST conversion rates for our cohort far exceed those for the general population (0.5%), as measured by using the same TST methods used by clerical workers of health care services in Turin between 1997 and 2004 (*30*).

The size of our cohort yielded the largest dataset on TB infection and disease among immigrants in Europe; this dataset allowed for stratified analyses, which indicated that different subgroups of socially marginalized immigrants carry considerably different risks for LTBI and TB. Knowledge of such differences is highly pertinent for determining which subgroups of socially marginalized immigrants should be screened and when the screening should be conducted. The decline in TST conversion rates some 6 years after immigration provides robust empiric evidence for concentrating screening of LTBI on immigrants within the first few years of arrival, as already recommended in several countries. Furthermore, the strong relationship between TB incidence in the country of origin and the risk for LTBI, active TB, and TST conversion prioritizes certain subgroups of socially marginalized immigrants for screening. The correlation of LTBI and TB prevalence with TB incidence rate in the immigrant’s country of origin probably reflects TB transmission that occurred in the countries of origin before immigration. By contrast, the increased risk for TST conversion, ≈20% for each increase of TB incidence rate in the country of origin of the immigrants of 50 cases/10^5^ person-years, reflects ongoing transmission in Italy after immigration.

Differences in TST conversion rates between immigrants originating from different countries are consistent with assortative mixing between immigrants from the same geographic area. Assortative mixing would favor the transmission of TB between immigrants from the same geographic area. As a result, immigrants from areas where the prevalence of TB is higher would be at a higher risk for TST conversion. Social contacts, mixing patterns, and health-related behaviors in the countries of origin before immigration may also explain the higher prevalence of TB and LTBI among men than among women (adjusted odds ratios were 1.5 and 1.1, respectively), a finding consistent with other published data (*31*).

In contrast with what has been observed among immigrants in other industrialized countries such as the United Kingdom, Denmark, and Canada, where the risk for TB decreases as a function of time elapsed since immigration (*1*,*2*,*4*), in our population the risk for active TB did not decrease with time since immigration. The reason for this unexpected finding is unclear but may be explained by an arising difference in exposure to conditions promoting LTBI reactivation. At entry, any socially marginalized immigrant must attend the screening program to have access to health and social care facilities. Over time since immigration, an increasing proportion of socially marginalized immigrants experience improvements in their socioeconomic condition and become easy to reach, and the attendant environmental changes, including nutrition and housing, might be expected to decrease their risk for LTBI reactivation. Such immigrants would no longer be required to be screened. The remaining long-term socially marginalized immigrants, who would continue to be screened, are persistently exposed to social and living conditions presumably favoring the reactivation of LTBI. Moreover, the occurrence of new infections, as measured through TST conversion, decreased with time elapsed since immigration regardless of the sex, age, and geographic origin of the immigrants. This decrease may represent a reduction of the transmission of *M. tuberculosis* among socially marginalized immigrants as a result of changes in contact patterns and difference of exposure resulting from the dilution of assortative mixing over time since immigration.

Our study has several limitations. The screening program was devised to reachthose persons accessing public and private health and social care facilities or the socially marginalized population less likely to be timely identified through routine passive case finding and contact tracing. For this reason, our target immigrant population is by definition socially marginalized and a credible denominator of immigrants cannot be determined; thus, we could not assess the coverage of screening program. Because the screening program does not collect detailed information on the reasons for immigration and for referral, we were unable to incorporate these factors in our analyses. Rates of completion of LTBI therapy after arrival in the destination for the study population are also unknown. LTBI prevalence and conversion rates were assessed by using TST rather than IGRAs, which became available in our setting in 2005 but for cost reasons have not hitherto been introduced for LTBI screening of immigrants. Our data would therefore tend to overestimate LTBI prevalence relative to data generated by IGRAs, which are more specific for LTBI (*11*,*16*,*32*); notwithstanding, the measures of association reported in Table 3 should not be affected, assuming that TST false-positive results are not differentially distributed across the variables investigated. Despite the relatively high specificity of TST conversion for recent infection, we cannot exclude the possibility that a fraction of the observed conversion is attributable to a boosted reaction as a result of repeated TST. To limit this possibility, we included in the analyses repeat TST performed not <12 months later (Figure 1). Estimated TST conversion rates may also have been affected by LTBI acquired by traveling to the countries of origin to visit friends and relatives (*33*), as suggested by the increased risk for TST conversion according to the TB incidence rate in the country of origin of the immigrants. On the other hand, the estimated reduction of conversion rates over time since immigration conflicts with this hypothesis. Overall, how much this hypothesis applies to the investigated immigrants, who are not representative of the general immigrant population, is uncertain. Finally, since reliable data on the BCG status of the socially marginalized immigrants were not available, we were unable to control for this in our analyses.

In conclusion, we have identified socially marginalized immigrants as a key reservoir of *M. tuberculosis* with substantial ongoing transmission in the first few years after arrival, which suggests that this population should be prioritized for screening for active TB and LTBI in countries where TB incidence is low. Our findings help to inform targeted interventions by identifying which immigrant subgroups should be prioritized for screening.

Technical Appendix Tables showing the available data, distribution of immigrants by geographic area of origin and age group, univariate model estimates of odds and incidence ratios, distribution, prevalence, and odds ratios for the prevalence of microbiologically confirmed tuberculosis cases in a study of *Mycobacterium tuberculosis* among socially marginalized immigrants in a low-incidence area of Italy.
