# Supplementary material for: High Rates of Mycobacterium tuberculosis among Socially Marginalized Immigrants in Low-Incidence Area, 1991–2010, Italy
Source: Emerg Infect Dis. 2013 Sep;19(9):1437–45. doi: 10.3201/eid1909.120200 (PMC3810899; doi:10.3201/eid1909.120200)
Supplement: Technical Appendix — Tables showing the available data, distribution of immigrants by geographic area of origin and age group, univariate model estimates of odds and incidence ratios, distribution, prevalence, and odds ratios for the prevalence of microbiologically confirmed tuberculosis cases in a study of Mycobacterium tuberculosis among socially marginalized immigrants in a low-incidence area of Italy. [file 12-0200-Techapp-s1.pdf]

Article DOI: http://dx.doi.org/10.3201/eid1909.120200

High Rates of *Mycobacterium tuberculosis* among Socially Marginalized Immigrants in Low-Incidence Area, 1991–2010

Technical Appendix

Technical Appendix Table 1. Set information included in the analyzed dataset in a study of high rates of *Mycobacterium tuberculosis* among socially marginalized immigrants in a low-incidence area, 1991–2010*

Variable	Note
Unique identification code†	Generated using the name, date and country of birth of each subject.
Sex	None
Country of birth	None
Date of birth	None
Date of immigration	None
Date of test	TST and chest radiograph if the examining physician suspected active pulmonary TB
TST result	Reported as millimetres of induration
Chest radiograph result	Reported as negative for TB or suspected active TB
Laboratory test result	Reported as microbiologically confirmed or not confirmed
Indicator of documented contact with an active TB case	Exclusion criterion

*TST, tuberculin skin testing; TB, tuberculosis.
†The identification was devised to preserve the individuality of each participant, while keeping the population anonymous to the authors of the paper.

Technical Appendix Table 2. Distribution of immigrants, by geographic area of origin and age group, in a study of high rates of *Mycobacterium tuberculosis* among socially marginalized immigrants in a low-incidence area, 1991–2010*

	No. (%) immigrants by age, y, group					
Geographic area of origin	0-19	20-29	30-39	≥40	Missing age	Total
Romania	446 (5.6)	2,853 (35.7)	2,432 (30.5)	2,043 (25.6)	212 (2.7)	7,986 (29.2)
Morocco	458 (11.0)	1,389 (33.3)	1,675 (40.2)	572 (13.7)	73 (1.8)	4,167 (15.2)
Sub-Sahara	202 (6.9)	1,470 (50.2)	958 (32.7)	256 (8.7)	45 (1.5)	2,931 (10.7)
Nigeria	130 (5.6)	1,519 (65.3)	596 (25.6)	65 (2.8)	17 (0.7)	2,327 (8.5)
Albania	577 (34.8)	641 (38.6)	296 (17.8)	129 (7.8)	16 (1.0)	1,659 (6.1)
Rep. OF Moldova	35 (2.1)	446 (27.1)	475 (28.9)	632 (38.4)	57 (3.5)	1645 (6.0)
Eastern Europe	139 (9.6)	463 (32.1)	371 (25.7)	434 (30.1)	37 (2.6)	1,444 (5.3)
Peru	102 (7.5)	483 (35.5)	478 (35.2)	261 (19.2)	35 (2.6)	1,359 (5.0)
Northern Africa	44 (4.4)	344 (34.0)	450 (44.5)	156 (15.4)	17 (1.7)	1,011 (3.7)
China	144 (17.1)	347 (41.2)	238 (28.2)	105 (12.5)	9 (1.1)	843 (3.1)
Latin America	78 (9.4)	314 (37.6)	305 (36.6)	126 (15.1)	11 (1.3)	834 (3.0)
Western Europe	57 (18.2)	95 (30.3)	105 (33.4)	45 (14.3)	12 (3.8)	314 (1.1)
Southeast Asia	12 (4.3)	93 (33.2)	114 (40.7)	52 (18.6)	9 (3.2)	280 (1.0)
South Asia	53 (20.3)	119 (45.6)	62 (23.8)	24 (9.2)	3 (1.1)	261 (1.0)
Middle East	18 (6.9)	120 (46.2)	91 (35.0)	29 (11.2)	2 (0.8)	260 (1.0)
North America	2 (8.0)	6 (24.0)	11 (44.0)	5 (20.0)	1 (4)	25 (0.1)
Oceania	1 (12.5)	3 (37.5)	2 (25.0)	2 (25.0)	0 (0)	8
Central Asia	2 (50.0)	0	0	2 (50.0)	0 (0)	4
Total	2,500 (9.1)	10,705 (39.1)	8,659 (31.7)	4,938 (18.0)	556 (2.0)	27,358 (100.0)

*Geographic areas reported do not include specific selected countries, which are reported individually.

Technical Appendix Table 3. Univariate model (logistic and Poisson) estimates of the odds and incidence rate ratios for TB, LTBI prevalence, and LTBI conversion in a study of high rates of *Mycobacterium tuberculosis* among socially marginalized immigrants in a low-incidence area, 1991–2010*

Variable	Odds ratio (95% CI) for prevalence		Incidence rate ratio (95% CI) for TST conversion
	TB	LTBI	
TB incidence rate/10^5 person-years in immigrants' country of origin			
<25	1 (ref)	1 (ref)	1 (ref)
25–49	2.5 (0.9–7.0)	2.1 (1.7–2.6)	1.1 (0.1–9.2)
50–99	4.9 (1.7–13.6)	1.7 (1.3–2.2)	1.3 (0.1–13.0)
100–299	4.6 (1.7–12.3)	3.0 (2.4–3.7)	3.2 (0.4–23.0)
≥300	3.5 (1.2–9.8)	2.4 (1.9–3.0)	3.5 (0.4–29.4)
Sex			
F	1 (ref)	1 (ref)	1 (ref)
M	1.4 (1.2–1.6)	1.1 (1.0–1.1)	0.9 (0.6–1.3)
Time, y, elapsed since immigration			
<3	1 (ref)	1 (ref)	1 (ref)
3–6	1.0 (0.8–1.2)	1.1 (1.0–1.2)	0.8 (0.4–1.4)
7–9	1.2 (0.9–1.6)	1.4 (1.2–1.5)	0.6 (0.3–1.0)
>10	1.2 (0.9–1.6)	1.4 (1.3–1.6)	0.4 (0.2–0.9)
Age, y, at test			
<20	1 (ref)	1 (ref)	1 (ref)
20–29	1.4 (1.0–2.1)	2.2 (1.9–2.4)	1.1 (0.4–3.3)
30–40	2.4 (1.7–3.5)	3.6 (3.2–4.0)	1.2 (0.4–3.2)
≥40	4.1 (2.8–5.9)	3.5 (3.1–4.0)	0.9 (0.3–2.8)

*The effects of the variables reported in the table were mutually adjusted. TB, tuberculosis; LTBI, latent TB infection; TST, tuberculin skin testing; ref, reference category.

Technical Appendix Table 4. Distribution and prevalence of microbiologically confirmed cases of tuberculosis in a study of high rates of *Mycobacterium tuberculosis* among socially marginalized immigrants in a low-incidence area, 1991–2010*

Variable	No. cases/no. population at risk	% (95% CI)
Total	512/27,358	1.8 (1.7–2.0)
Sex		
F	181/11,116	1.6 (1.4–1.9)
M	331/16,242	2.0 (1.8–2.3)
Age, y, at test		
<20	25/2,498	1.0 (0.6–1.5)
20-29	135/10,705	1.3 (1.0–1.5)
30-39	166/8,659	1.9 (1.6–2.3)
≥40	186/5,414	3.4 (3.0–3.9)
Background TB incidence groups, no. cases/10^5 person-years		
<25	3/679	0.4 (0.09–1. 3)
25-49	40/3,500	1.1 (0.8–1.5)
50-99	27/1,668	1.6 (1.1–2.3)
100-299	417/20,050	2.1 (1.9–2.3)
≥300	25/1,461	1.7 (1.1–2.5)

*TB, tuberculosis

Technical Appendix Table 5. Odds ratio for TB prevalence (95% CI). Analysis restricted to microbiologically confirmed cases of TB*

Variable	Univariate logistic regression model	Multivariate logistic regression model
TB incidence rate/10^5 person-years in immigrants' country of origin		
<25	1 (ref)	1 (ref)
25–49	3.3 (0.8–13.9)	3.2 (0.8–13.2)
50–99	5.2 (1.2–22.1)	4.7 (1.1–20.0)
100–299	5.9 (1.5–23.6)	5.2 (1.3–20.1)
≥300	4.8 (1.1–20.4)	5.1 (1.1–21.6)
Sex		
F	1 (ref)	1 (ref)
M	1.2 (1.0–1.5)	1.4 (1.1–1.6)
Time, y, elapsed since immigration		
<3	1 (ref)	1 (ref)
3–6	0.9 (0.7–1.2)	1.0 (0.8–1.3)
7–9	1.2 (0.9–1.6)	1.1 (0.8–1.5)
>10	1.2 (0.8–1.8)	0.9 (0.6–1.4)
Age, y, at test		
<20	1 (ref)	1 (ref)
20–29	1.3 (0.8–2.0)	1.2 (0.8–1.9)
30–40	1.9 (1.3–3.0)	1.8 (1.1–2.8)
≥40	3.5 (2.3–5.5)	3.4 (2.2–5.2)

*The effects of the variables reported in the table were mutually adjusted. TB, tuberculosis; ref, reference category. TB, tuberculosis
